# Red, Purple and Pink: The Colors of Diffusion on Pinterest

**DOI:** 10.1371/journal.pone.0117148

**Published:** 2015-02-06

**Authors:** Saeideh Bakhshi, Eric Gilbert

**Affiliations:** 1 HCI Research Group, Yahoo Labs, San Francisco, California, United States of America; 2 College of Computing, Georgia Tech, Atlanta, Georgia, United States of America; University of Melbourne, AUSTRALIA

## Abstract

Many lab studies have shown that colors can evoke powerful emotions and impact human behavior. Might these phenomena drive how we act online? A key research challenge for image-sharing communities is uncovering the mechanisms by which content spreads through the community. In this paper, we investigate whether there is link between color and diffusion. Drawing on a corpus of one million images crawled from Pinterest, we find that color significantly impacts the diffusion of images and adoption of content on image sharing communities such as Pinterest, even after partially controlling for network structure and activity. Specifically, Red, Purple and pink seem to promote diffusion, while Green, Blue, Black and Yellow suppress it. To our knowledge, our study is the first to investigate how colors relate to online user behavior. In addition to contributing to the research conversation surrounding diffusion, these findings suggest future work using sophisticated computer vision techniques. We conclude with a discussion on the theoretical, practical and design implications suggested by this work—e.g. design of engaging image filters.

## Introduction

Color is a ubiquitous perceptual stimulus that is often linked with psychological functioning in humans [1]. For example, in 1979, a director at the American Institute for Biosocial Research began observing curious psychological variation in his patients—variation seemingly rooted in what colors he showed them. To test his theory, he convinced the directors of a naval prison to paint their cells pink, believing pink would calm the inmates. What he found was fascinating. Rates of violent behavior fell dramatically after exposure to the plain pink walls. According to the Navy’s follow-up report, "Since the initiation of this procedure … there have been no incidents of erratic or hostile behavior" [2].

In addition to inducing calm, colors can evoke powerful reactions like warmth, relaxation, danger and energy [3–6]. In short, they have remarkable power to move us emotionally. For example, prior work has shown that Red is associated with excitement, Yellow with cheerfulness and Blue with comfort [7]. In this paper, we ask: Might these phenomena documented in lab experiments also affect online behavior? Could color drive how we act on social media?

Recently, we have seen image-sharing communities truly take off—sites such as Pinterest, Imgur and Tumblr, just to name a few. A key research challenge for communities like these is uncovering the mechanisms by which content spreads from person to person (or “diffuses,” adopting the term from the academic literature). For example, a study of the most widely shared New York Times stories found that they tend to “inspire awe” in their readers [8]. While we have results like this for text and network structure (e.g., Bakshy et al. [9] and Sun et al. [10]), as far as we know, we have no such similar results on what makes images diffuse widely. It is within this context that we make the leap from color to diffusion: Is there a link between them?

In this paper, we aim to answer whether color stimulations affect behavior online. We adopt Pinterest as our research site. Pinterest is a rapidly growing social network based on images. Drawing on a corpus of one million images crawled from Pinterest, we find that color significantly drives how far an image diffuses (to what extent it is adopted by other users), even after partially controlling for user activity and network structure. Specifically, Red, purple and pink seem to promote diffusion, while Green, Blue and Yellow suppress it. As far as we know, this is the first result describing how image features affect diffusion. Our work bridges the gap between online user behavior and psychology studies of color. In addition to contributing to the ongoing research conversation surrounding diffusion, we believe these findings suggest future research to uncover the impact of color on other aspects of online user behavior, and to use more sophisticated computer vision techniques. For example, could we apply advanced computer vision techniques to relate an image’s macro-properties (outside vs. inside, natural vs. cityscape) to the image diffuses?

For designers, our findings shed light on engagement on large social sites. Recently, for example, many mobile applications let users transform their photos with image filters. Instagram popularized this technique, but similar features can be found in Flickr’s latest mobile app. The filters typically change saturation, brightness, and color distributions. Our results can be used to guide the design of these filters. For example, filters that increase an image’s saturation or enhance its warmness maybe likely to increase diffusion—a highly sought-after form of engagement.

## Related Work

We describe modern literature on content characteristics and social diffusion online. We also summarize previous work on images and social behavior around them online. We then turn to prior research on the effects of color and its associations with emotions and choice.

### Content Characteristics and Social Diffusion

Ellison and colleagues note that “the primary function of these [social network] sites is to consume and distribute personal content about the self” [11]. Sharing content can in turn ensure that users remain engaged and committed in the future [12]. Users have diverse motivations to share content on social network sites [13]. While the focus of most of these studies is largely network structure, content can, of course, also be the reason behind diffusionccccbakshy2011everyone. Users may share useful content to appear knowledgable or simply to help out [14]. The emotional valence behind content can also drive its sharability. For example, Jamali et al. used a Digg dataset to predict the popularity of stories [15], where sentiment emerges as a major predictor. In another study, Berger et al. used New York Times articles to examine the relationship between the emotion evoked by content and virality [8], and found that stories that inspire awe in readers get shared the most.

Several recent empirical and theoretical research papers have studied diffusion in various social networks. Examples include studies on Facebook diffusion trees [10], diffusion of gestures between friends on Second Life [9], diffusion of health behavior [16] and adoption of mobile phone applications over the Yahoo! messenger network [17]. In most of these studies, network structure is considered to be the main driving cause of influence and diffusion. Influence is largely regarded as the ability to cause diffusion. For instance, a study by Kwak et al. compared three network measures of influence: number of followers, PageRank, and number of retweets [18]. Cha et al. also considered an additional measure, number of mentions on Twitter, to quantify diffusion [19].

When it comes to visual analysis of content of photos, there is little existing scholarly work. In a recent piece, Hochman et al. analyzed colors in photos uploaded to Instagram from two different cities of New York and Tokyo and found differences across the two locations [20]. For instance, hues of pictures in New York were mostly Blue-Gray, while those in Tokyo were characterized by dominant Red-Yellow tones. In an earlier work, we studied the engagement value of photos with human faces in them [21]. We found that photos with faces are more likely to receive likes and comments.

This work builds on previous work on social diffusion, and engagement in particular. It takes a new perspective by considering the color as a stimulus of online social behavior.

### Color as affective stimulus

Given the ubiquity of color in our lives, it’s not surprising that a great deal of research has been conducted over the past century on it. Scientists widely recognize color as a source of impact on our emotions and feelings [3–6]. Color is believed to affect the degree of felt arousal [22, 23]. This view of arousal has been predominant in both psychology [24] and marketing [25, 26].

Of the theoretical based research, Goldstein’s work conceptualized colors and psychological functioning around colors [27]. Many theoretical researchers have followed this conceptualization. For example, Apter and his colleagues used a two dimensional theory to explain arousal and pleasure in color research [28, 29]. According to their theory, there are two dimensions of arousal: one goes from boredom to excitement, the other goes from tension to relaxation. Both excitement and tension can produce equivalently high states of arousal, but the former would be pleasant while the latter would be unpleasant [23]. Using the two-dimensional view of arousal, they discovered a link between Red and felt excitement, and Blue and felt relaxation (also see related work [30, 31]).

Several empirical projects have studied the role of color in affective marketing. One stream of research have examined the specific colors used in magazine ads [32, 33]. The second stream of research has investigated the efficiency of colors compared with Black and White ads [34, 35]. The third stream has focused on the effects of specific colors on consumer responses [36, 37]. This line of work suggests, for example, that Red backgrounds elicit greater feelings of arousal than Blue ones, whereas products presented against Blue backgrounds are liked more than products presented against Red ones [36, 38]. Increases in arousal are at first pleasurable and exciting, but after a certain point, more will decrease pleasure and increase tension [24].

Color theorists believe that color also influences cognition and behavior through learned associations [39]. Color can affect cognitive task performance, with Red and Blue activating different motivations and consequently affecting different types of tasks [40]. In another article, researchers studied the relationship of color and emotion among college students by asking them about the feelings colors brought to mind [4]. They found that the principal hues showed the most positive emotional responses, followed by intermediate hues and achromatic colors.

In addition to hues, studies have examined saturation and brightness as stimuli. More deeply saturated colors can be more exciting and cause surprising behavior [41, 42]. Other research supports the idea that higher levels of chroma are more widely liked [30, 43–45]. Another study concludes from experiments that hue, chroma and value are linked to consumers’ feelings and shopping attitudes: Higher levels of saturation seem to increase excitement, while increases in brightness lead to feelings of relaxation [46].

Previous work suggests that color can impact emotions, choices and behavior. In this research, we ask whether such effects are observable online as well.

### Specific colors and their associations

Prior psychology studies argued that colors are associated with certain abstract concepts. Marketing psychologists suggested that a sustained color impression is made on a subject within 90 seconds and that color accounts for 60% of the acceptance or rejection of an object, place, individual or circumstance [47]. Because color impressions are made quickly and are long lasting, decisions regarding choice of color can be highly important to marketing success [48].

In previous research, Blue is associated with wealth, trust and security [7]; Gray is associated with strength, exclusivity, and success; and, Orange connotes cheapness [49]. Green is seen as cool, fresh, clear and pleasing, but when illuminated on skin tones it becomes repulsive or can be associated with tiredness and guilt [6, 50, 51]. In a recent study, Kuhbandner and Pekrun [52] found that the effects of emotion on memory depended on color type. Red strongly increased memory for negative words, whereas Green strongly increased memory for positive words. Additionally, researchers found that a brief glimpse of Green prior to a creativity test could enhance creative performace [53].

Red is known as a dominant and dynamic color, but this can have both positive and negative effects. Previous research has shown that Red can enhance human performance in contests [54] and detailed-oriented tasks [40], it can lead men to view women as more attractive and sexually desirable [55], and at the same time; it can induce avoidance motivation [40]. Purple is mostly associated with children and laughing, a positive association reported by Kaya et al. [4]. Psychological studies suggest that White has a calming effect, producing the least amount of tension [56, 57]. The implication is that higher value, lighter colors should be more relaxing than lower value, darker colors. The well-known designer, Faber Birren, claimed that Blue, Red, Grey, Orange and Yellow color preferences are nearly identical for both sexes and exist beyond cultural boundaries [48].

Our work builds on this research by looking for the first time at diffusion as a function of color stimulus. We connect the anecdotal, experimental and theoretical studies of color with the spread of online content.

## Methods

We take a quantitative approach in this paper to investigate how color shapes diffusion. In this section, we first describe the data we collected and our statistical methods. We adopt the Munsell color system to identify and categorize colors; it is a widely used system in psychology and physiological studies of color.

### Ethics statement

We collected our dataset by crawling Pinterest using the publicly available pages on the site. The dataset is publicly available at https://bitbucket.org/compsocial/2014-pinterest-data. Pinterest has granted us permission to use, analyze and share this dataset.

### Data

We collect our data from Pinterest, a pinboard-style, photo-sharing social network site that allows users to upload images or bookmark them from external websites. Pinterest is the fastest growing website in the web’s recent history, with 429% growth from September to December 2011 [58].

**Figure 1:**
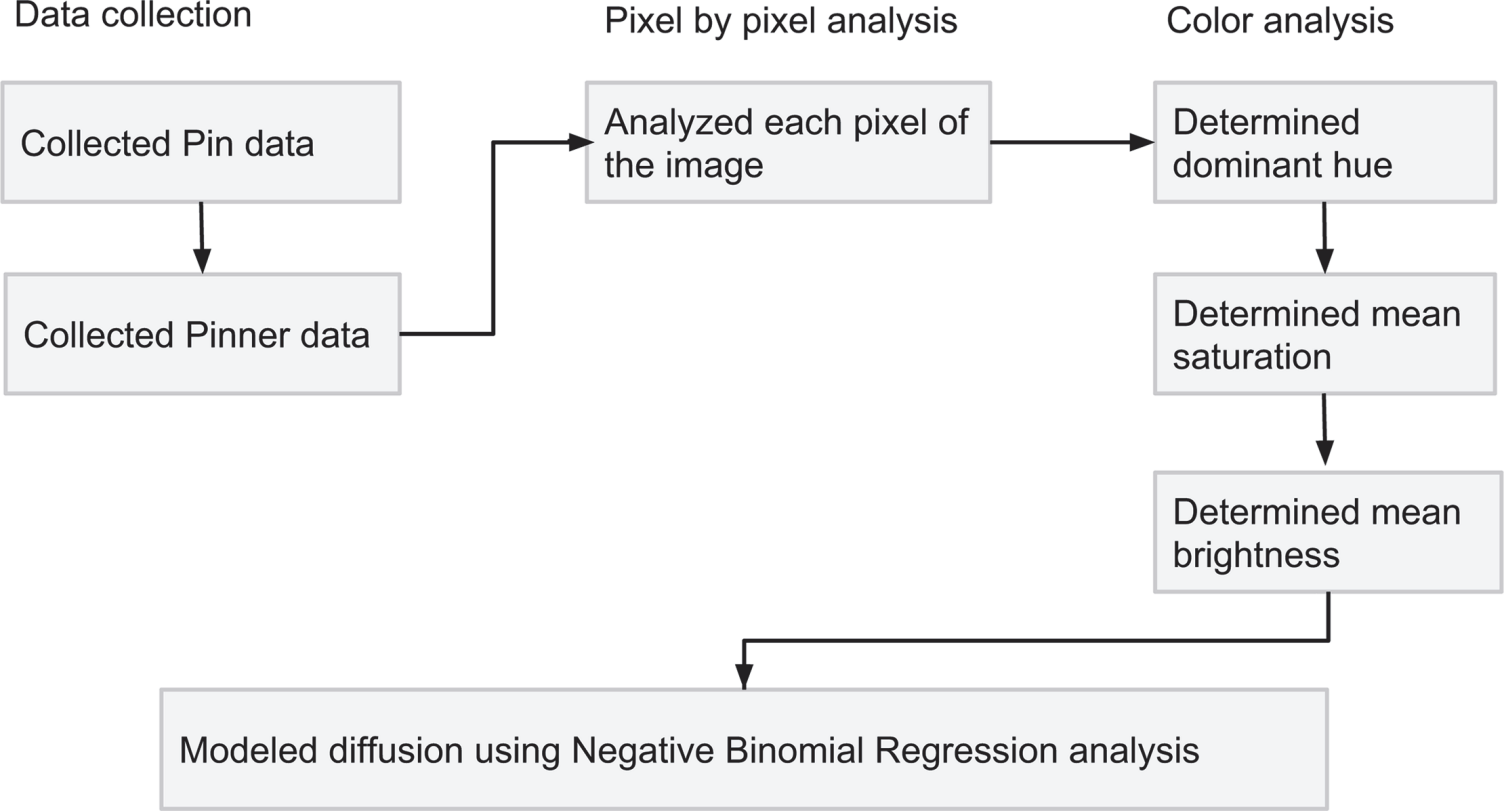
A flowchart of steps taken in this paper to prepare data for analysis. We collected a random set of pins from Pinterest. Each pin’s image was analyzed pixel by pixel, resulting in a dominant hue, mean saturation and mean brightness. Negative Binomial Regression was used to characterize the effects of colors on repins.

While our goal was to obtain a random sample of pins and pinners, there is no publicly available means to do so. Instead, we developed a web crawler to approximate a random sample [59]. Using this method, we collected a total of one million pins and their associated meta-data. We also collected the pinners for these pins, ending up with a set of 989,355 pinners. The final dataset is uniformly distributed across months of the year and does not show any seasonal bias. Data spans all 12 months of 2009–2011 collected in June 2012. Fig. 1 presents an overview of the the data collection and analysis process. Table 1 summarizes the basic statistics of the variables used in this paper. We describe our dependent, control and color variables in detail below.

**Table 1:** Summary statistics of quantitative variables used in this paper.

Variable	Min	μ	x̃	σ	Max
pinner pins	0	99.4	0	798.15	55,730
followers	0	6.36	0	51.15	1,915
repins	0	1.45	0	35.66	15,180
mean saturation	0	0.31	0.29	0.18	1
mean brightness	0	0.63	0.64	0.17	1

Response Variable (Dependent measure). We use the *number of repins* (i.e., the number of times the pin was shared) as our dependent variable in this paper. Repin is our measure of the pin’s diffusion.

Predictor Variables. We group our predictor variables into two categories: the control features and the color features. We are interested in the effect of color features on the response measure. We use control features to compare the effect of colors

**Control features**. A pin is composed of an image (or sometimes a video) that links to content outside Pinterest. Users can upload their images or use Pinterest’s bookmarklet on other websites to create a pin. All pins on Pinterest link back to their source and they can be repinned by other users. Pinterest users can organize their pins by topic into self-defined boards. Users can set boards to private or public, and the boards may attract followers independent of the user’s other boards.

On Pinterest, users generate social networks based on “follow” relationships. When A follows B, B’s pins will show up in A’s Pinterest feed. The “following” relationship can be limited to certain boards, so that users don’t get updates from all pins. We are interested in some user features that signal activity and social network reach. For activity, we use the *number of pins* on a user’s profile. The *number of followers* is our measure of the user’s influence. This is a powerful and intuitive control, as we expect pinners with larger audience to have a higher baseline probability of being repinned by their followers. Table 1 lists basic statistics of these variables.

**Color features**. Images are the focal point of our study. For every pinned image, our code traverses each pixel and extracts RGB values. We convert the RGB space to HSV space to be able to describe the actual color in a more human perceivable form. We use the following equations to convert the RGB values to the corresponding HSV system. To perform the conversion, we first normalize the R, G, B values by dividing them by 255 to change the range to [0,1]. We then calculate *C_max_*, *C_min_* and Δ as following:

$$C_{max}=max(R,G,B)$$

$$C_{min}=min(R,G,B)$$

$$\Delta =C_{max}-C_{min}$$

We can then calculate Hue as following:

$$H=\left\{ \begin{array}{l} 60^{\circ }\times (\frac{G-B}{\Delta }\text{mod}\;6)\;,C_{max}=R\\ 60^{\circ }\times (\frac{B-R}{\Delta }+2)\;,C_{max}=G\\ 60^{\circ }\times (\frac{R-G}{\Delta }+4)\;,C_{max}=B \end{array}\right.$$

We compute saturation for each pixel using the following equation:

$$S=\{\begin{array}{cc} 0 & ,\Delta =0\\ \frac{\Delta }{C_{max}} & ,\Delta \neq 0 \end{array}$$

and value or chroma using the following equation:

$$V=C_{max}$$

Although commonly used, the HSV system does not ensure that hues are equated on chroma and value. Having HSV values for each pixel in the image, we used the Munsell color system to map each pixel to one of the ten major hues (The Munsell system is described in greater detail next.). We then find the most dominant color in the image (mode of the distribution) and use it in our model. Choosing the dominant color as the main representative color of an image might have limitations. For example, if the distribution of hue in the image has multiple modalities, perceiving a single color as the most dominant might be difficult. We perform a human validation experiment on Mechanical Turk to make sure this is not a fundamental issue with our dataset.

We calculate the mean saturation and brightness of the image and use them as predictors for repin model. The saturation and brightness are on a scale of 0 to 10 in Munsell Color System (see next section). For simplicity, we convert saturation and brightness to a scale of 0 to 1 in this paper. Table 1 summarizes the basics statistics of saturation and brightness for our dataset.

In addition to the 10 major hues, we identified the images which were mostly consisting of White or Black. We also used a binary feature which identifies whether the image is only consisted of Black and White colors. If the pin’s image is Black and White this variable is set to 1. While the main goal of this work is to identify color properties that might affect diffusion of content, we have to be careful with the content and topic of images that are posted on Pinterest. Table 2 summarizes some of the major topics photos on Pinterest belong to and the distribution of photos by dominant hue categories. We see that Yellow, Red-Yellow and Yellow-Green are common dominant hues among most categories. While the distributions differ from one category to another, we did not find any statistical significance. We performed Pearson’s Chi-square test between each category and the the photos that are not assigned to any categories.

**Table 2:** Distribution of dominant hues across each category of content.

Category	Red	Red Yellow	Yellow	Yellow Green	Green	Green Blue	Blue	Blue Purple	Purple	Purple Red
animals	9.0%	20%	28%	20%	11.7%	6.3%	2.7%	1.0%	0.7%	0.0%
architecture	2.6%	16%	32%	23%	14%	6.7%	4.1%	0.0%	0.0%	0.5%
art	9.1%	16%	28%	21%	14%	8.2%	2.6%	0.0%	0.0%	0.0%
celebrities	21%	21%	19%	15%	12%	4.8%	3.3%	1.3%	0.6%	0.6%
design	2.2%	24%	28%	22%	11%	5.7%	4.6%	1.1%	0.3%	0.0%
DIY crafts	1.5%	18%	25%	23%	16%	9.7%	3.6%	1.9%	0.7%	0.3%
education	5.6%	21%	22%	19%	17%	8.1%	3.9%	1.0%	1.4%	0.5%
film, music, books	21%	19%	17%	17%	11%	6.1%	5.5%	1.0%	1.0%	0.3%
food, drink	1.0%	28%	34%	20%	9.8%	4.1%	1.7%	1.0%	0.2%	0.1%
gardening	1.1%	11%	27%	32%	20%	6.2%	1.7%	1.0%	0.0%	0.0%
hair, beauty	3.3%	31%	27%	19%	10%	4.9%	2.7%	0.0%	0.4%	0.0%
health, fitness	3.9%	23%	26%	23%	9.5%	8.5%	3.9%	2.0%	0.0%	0.7%
holidays events	1.0%	22%	26%	21%	15%	7.4%	4.4%	2.5%	0.5%	0.0%
home decor	1.6%	26%	30%	24%	11%	5.2%	1.1%	1.0%	0.2%	0.1%
humor	8.2%	21%	21%	18%	11%	6.2%	5.0%	4.2%	2.8%	1.0%
illustrations, posters	18%	24%	18%	13%	11%	8.2%	2.7%	1.1%	1.4%	1.1%
kids	2.9%	18%	26%	22%	18%	7.8%	3.2%	1.5%	0.2%	0.0%
outdoors	0.0%	12%	31%	21%	18%	12%	5.2%	0.0%	0.0%	0.0%
photography	16%	21%	22%	16%	13%	7.2%	3.4%	1.1%	0.0%	0.2%
products	8.6%	27%	25%	14%	14%	7.4%	1.7%	1.7%	0.0%	0.0%
travel	3.3%	13%	21%	21%	20%	15%	4.8%	0.9%	0.0%	0.2%
weddings	3.9%	27%	27%	22%	11%	5.4%	1.8%	0.9%	0.2%	0.1%
womens fashion	4.6%	27%	28%	18%	12%	6.6%	2.5%	0.9%	0.1%	0.1%
other	5.9%	23%	24%	19%	13%	7.5%	4.1%	1.3%	1.0%	0.4%
all categories	4.2%	24%	27%	20%	12%	6.6% 2.8%	1.1%	0.4%	0.2%	

### The Munsell System

Color representations allow us to specify or describe colors as a low-dimensional projection—useful for meaningful modeling. We usually identify colors with simple names, but names can also be subjective or culture-dependent. In this paper, we use the Munsell system [60] to characterize colors, a widely used system in applications requiring precise specification of colors, including psychological studies [41]. According to this system, each color has three basic attributes: hue, chroma (saturation) and value (brightness).

**Figure 2:**
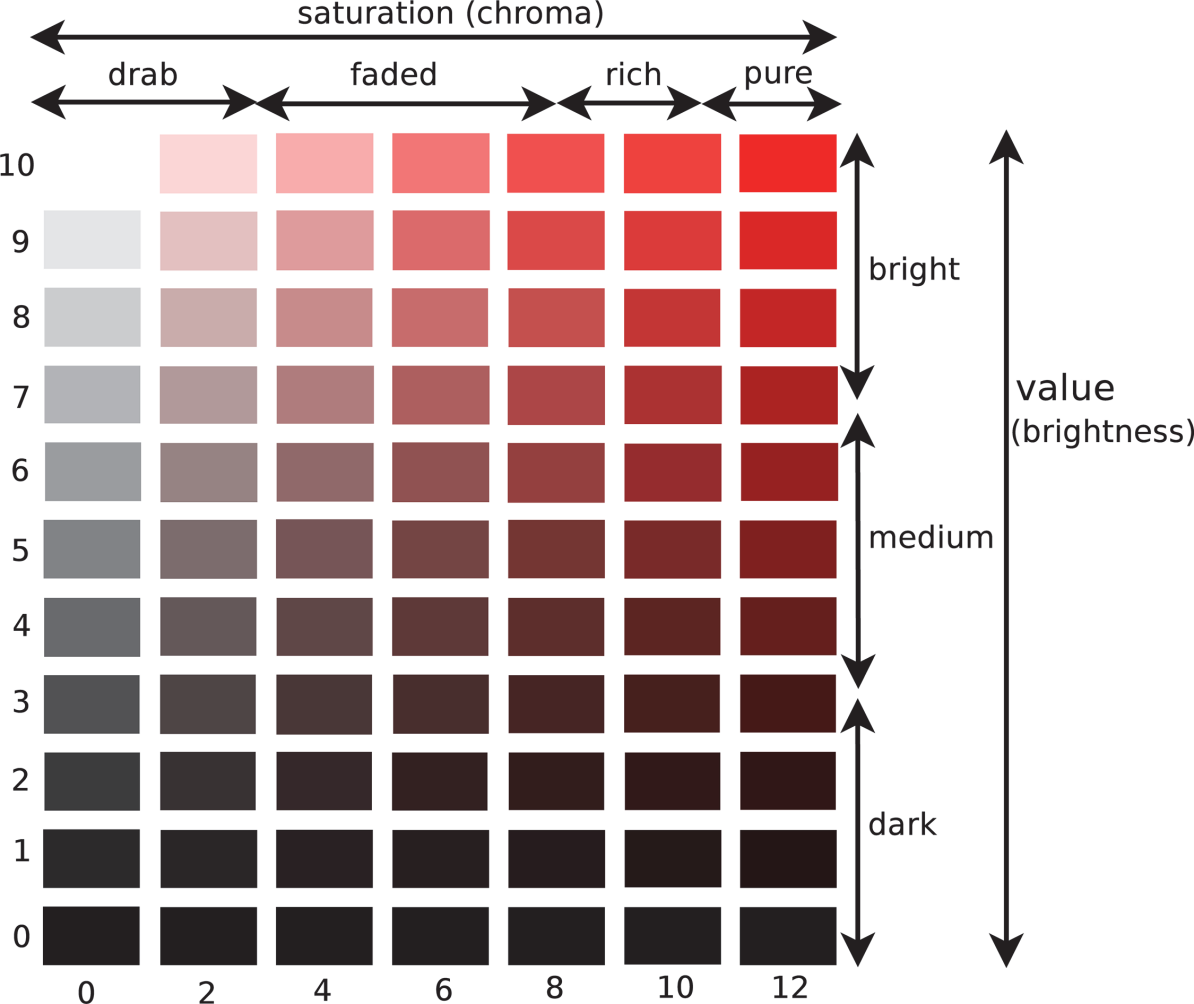
The Munsell color system specifies colors based on hue, chroma (saturation) and brightness (value). We used this system to classify color of images in this paper. The left image shows the combinations of brightness and chroma for the color Red. The right image illustrates how the Munsell system divides the hue space into 10 different colors.

Hue is a color’s pigment, what we normally understand as Blue, Red, Yellow, etc. There are ten hues in the Munsell color system, five of which are identified as principal hues (i.e., Red, Yellow, Green, Blue, and Purple). The other five colors are the intermediate hues (Red-Yellow, Yellow-Green, Green-Blue, Blue-Purple and Purple-Red). Fig. 2 demonstrates the classification of ten different hues.

Chroma refers to saturation, the degree of purity or vividness of the hue. Highly saturated colors have higher proportions of pigment in them and contain less Gray. Low chroma colors are drab and dull, while the high chroma colors are rich and pure. Brightness, on the other hand, refers to the degree of darkness or lightness of the color. Low brightness colors look *blackish* while high brightness colors look *whitish*. In the Munsell system, a brightness of 0 is used for pure Black, while a brightness of 10 is used for pure White. Black, White and the shades of Gray are called neutral (achromatic) colors. Fig. 2 illustrates all three dimensions of color. The leftmost image shows saturation versus brightness, while the right demonstrates hue classification.

### Statistical Methods

Our dependent variable, the number of *repins*, is a *count* variable. We model the number of repins using *Negative Binomial* regression on two classes of independent variables: control atrributes and color attributes. Negative binomial regression is well-suited for *over-dispersed* distributions of *count* dependent variable [61]. We use negative binomial regression instead of *Poisson* regression since the variance of the dependent variable is larger than the mean (*μ* = 1.45, *σ* = 35.66). We use over-dispersion to test whether *Poisson* or *Negative Binomial* regression should be used. This test was suggested by Cameron and Trivedi [61], and involves a simple least-squares regression to test the statistical significance of the over-dispersion coefficient.

The *Negative Binomial* regression models the *expected* number of repins *y* for an image as a function of control and color independent variables. We construct two regression models to evaluate the *impact* of control and color variables: first to model control variables alone (ctrl model), and the second to model both control and color variables (ctrl+ clr model). The reduction in deviance from the full model to the control-only model shows the significance of color variables on explaining the number of repins.

The first model uses control attributes (ctrl) as predictors of the number of repins an image receives.

$$ln(y)=I+\Sigma_{i}^{x_{i}\in ctrl}\beta_{i}x_{i}$$ (1)

where *I* is the intercept for the model and the control sum is computed using the following network structure and activity attributes:

$$\begin{array}{l} \Sigma_{i}^{x_{i}\in ctrl}\beta_{i}x_{i}=\beta_{\text{followers}}x_{\text{followers}}+\beta_{\text{pins}}*x_{\text{pins}} \end{array}$$ (2)

This model allows us to understand the effect on the number of repins of control variables alone. We then model the impact of color factors (hue, saturation and value) on the number of repins as follows. We construct a second model that includes both control attributes and color attributes as predictors.

$$ln(y)=I+\Sigma_{i}^{x_{i}\in ctrl}\beta_{i}x_{i}+\Sigma_{j}^{x_{j}\in clr}\beta_{j}x_{j}$$ (3)

where, the control sum is taken from Equation 2 and the color sum is computed using color-related variables:

$$\begin{array}{l} \Sigma_{j}^{x_{j}\in clr}\beta_{j}x_{j}=\beta_{\text{dominantHue}}x_{\text{dominantHue}}++\beta_{\text{isBlack\&White}}x_{\text{isBlack\&White}}\\ +\beta_{\text{meanSaturation}}x_{\text{meanSaturation}}+\beta_{\text{meanValue}}x_{\text{meanValue}} \end{array}$$ (4)

Here, *x*_dominantHue_ is the categorical variable for dominant identified hue in the image, *x*_meanSaturation_ is a numerical variable quantifying the mean saturation across all pixels in the image, *x*_meanValue_ is a numerical variable quantifying the mean value across all pixels in the image and *x*_isBlack&White_ is a binary variable identifying whether the image is considered Black and White or not.

The regression coefficients *β* allow us to understand the effect of an independent variable on the number of repins (note that to be able to compare coefficients, we z-score all numerical variables before performing regression). In order to choose which subset of independent variables should be included in the number of repins model, we use the *Akaike Information Criterion (AIC)* [62]. AIC is a measure of the relative quality of one model against another, and is defined as following:

AIC=−2L+2k

where, *k* is the number of parameters and *L* is the maximum log-likelihood of the model. The smaller the value of AIC, the better the fit of the model. Starting with a full set of independent variables (image features, number of pixels, color features, etc.), we use a step-wise procedure to select the model that minimizes AIC. Using the model with minimum AIC also reduces the chances of choosing a model that overfits the data.

We test coefficients of all independent variables for the null hypothesis of a zero-valued coefficient (two-sided). This method is based on standard errors of coefficients, which is analogous to the *t-test* used in conventional regression analyses. We use a Chi-square test with one degree of freedom to test the hypothesis that each coefficient *β_j_* is zero. To do this, we compute the following term:

$$\chi^{2}=\frac{b_{j}^{2}}{{\left(SE_{j}\right)}^{2}}$$

where, *b_j_* is the estimate of *β_j_* and *SE_j_* is the standard error of the coefficient *β_j_*. Table 3 shows the *β* coefficients and the *p* -values from the Chi-square test. We see that almost all independent variables (and interaction variables) have coefficients that are statistically significant.

**Table 3:** The results of negative binomial regression with number of repins as the dependent variable.

type	Variable	β	Pr(>|z|)
Controls	pinner followers	0.82	<10^−15^
	pinner pins	−0.76	<10^−15^
Color	saturation	0.25	<10^−8^
	brightness	0.02	<10^−12^
	Red	0.49	<10^−15^
	Red-Yellow	0.02	<10^−4^
	Yellow	−0.08	<10^−4^
	Yellow-Green	−0.10	<10^−12^
	Green	−0.13	<10^−8^
	Green-Blue	−0.01	<10^−4^
	Blue	−0.17	<10^−12^
	Blue-Purple	0.03	<10^−8^
	Purple	0.31	<10^−12^
	Purple-Red	0.16	<10^−15^
	Black	−0.12	<10^−11^
	White	0.04	<10^−15^
	Black and White	−0.12	<10^−8^
	(Intercept)	0.47	<10^−15^

The model is significant and reduces 55% of deviance.

We use the deviance goodness of fit test to assess our regression fit [63]. The deviance is expressed as:

$$D=2\sum_{i=1}^{n}(\zeta (y_{i};y_{i})-\zeta (\mu_{i};y_{i}))$$

with ζ(*y_i_; y_i_*) indicating a log-likelihood function with every value of *μ* given the value *y* in its place. The ζ(*μ_i_; y_i_*) is the log-likelihood function for the model being estimated. The deviance is a comparative statistic. We use the *Chi-square test* to find the significance of the regression model, with the value of deviance and the degrees of freedom as two Chi-square parameters. The degrees of freedom is the number of predictors in each model. Table 4 summarizes the model parameters and the goodness of fit test results, and shows that the regression models are a good fit for our data.

**Table 4:** Summary of the ctrl and ctrl+clr models for the number of repins.

Model	θ	Resid. df	2 x log-lik.
ctrl model	0.16	850599	−3158102.61
ctrl+clr model	0.20	850205	−3144231.54
**Summary**			
LR.stat			13871.07
degrees of freedom			11
Pr(> Chisq)			<2e^−12^

The Chi-square test on the difference between deviances in the models shows significance of ctrl+clr model compared to ctrl only model.

### Dominant Color Validation

As we described in the previous section, we used a pixel-based method to find the most dominant color in the image: the algorithm picks the modal hue class. To make sure that the dominant color found in the image via this method matches what people actually perceive, we perform an evaluation experiment on Mechanical Turk.

**Figure 3:**
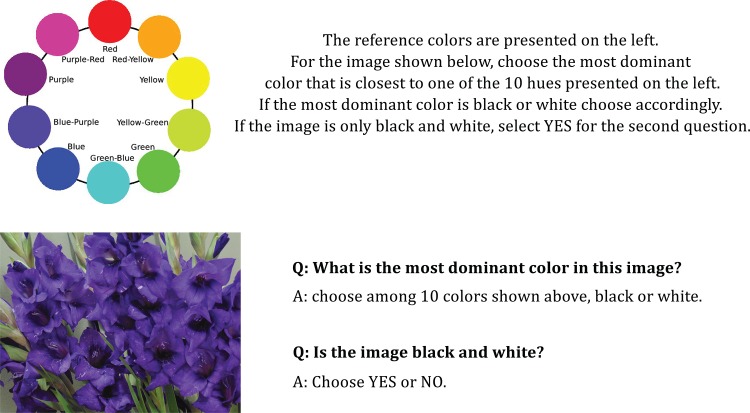
A sample Mechanical Turk task for evaluating our color extraction method. The example image in this figure was taken from Flickr Creative Commons collection: http://flic.kr/p/o8La3U.

We randomly selected a subset of 2,000 images from our dataset and employed 20,000 Mechanical Turk workers to validate our dominant hue identification algorithm. We pre-screened turkers to make sure they are not color-blind. We asked turkers to identify the dominant hue they perceived. We also asked them to mark the image as Black-and-White if they recognize it as a Black-and-White image. We provided them with the Munsell color chart as shown in Fig. 2 and asked workers to choose the closest hue in the Munsell system that matches the dominant color in the image. Fig. 3 shows a sample task delivered to Turkers.

All 2,000 images were evaluated by at least five Turkers. We recorded the perceived dominant color as the one that all participants agreed on. In every single case, at least three Turkers agreed on a dominant hue. We had to remove 18 (less than 1%) of the images due to inconsistency between Turkers’ responses. We then compared the dominant color identified by Turkers with the one our algorithm found. Of the 1,982 in the test set, 1,880 of the images (95%, margin of error 0.51) were in agreement with our algorithm’s decision. While the 5% disagreement rate introduces some noise into the subsequent statistical models we present, we were convinced by this high level of agreement to move onto large-scale application of the dominant hue algorithm. Table 5 shows the percentage of dominant hues validated in each hue class, with the associated margin of error.

**Table 5:** Results of mechanical turk evaluation for dominant hue detection.

Dominant Hue	Sample size	Correct %	MOE
Red	246	91%	2.01%
Red-Yellow	145	98%	1.19%
Yellow	125	97%	1.6%
Yellow-Green	136	96%	1.81%
Green	212	94%	1.65%
Green-Blue	139	94%	2.06%
Blue	239	94%	1.58%
Blue-Purple	193	96%	1.37%
Purple	118	92%	2.6%
Purple-Red	126	92%	2.57%
Black	87	94%	2.61%
White	98	92%	2.97%
Black and White	118	91%	2.76%

Margin of Error (MOE) is computed for 95% confidence.

## Results

Table 3 summarizes the *β* coefficients of the *Negative Binomial* regression model for repin counts. We use the Chi-square Test to find the significance of the regression model, by computing the reduction in deviance from a *null model*. For our model for the repins, we found the reduction in deviance *χ* ^2^ = 3.1M—1.42M, or a 55% drop, on 17 degrees of freedom. The test rejected the null hypothesis, *p* <10^−15^; hence, the regression model is well-suited to characterize the effects of the independent variables.

### Control Effects

As expected, a pinner follower count has a large positive effect on the virality of a pin, *β* = 0.82, *p* <^−15^. This means the higher the number of followers, the more likely it is for the pin to get repinned. On the other hand, the number of pins someone has on his/her boards shows a negative effect on repins, *β* = −0.76, *p* <^−15^. The number of pins is an indicator of activity on Pinterest. As we can see in our results (Table 3) the higher the activity (number of pins) the lower chances of receiving repins. This might also be interpreted another way: the more pins a pinner has, the lower probability any single one has of being repinned. This predictor acts as a drag coefficient in our model.

As expected, we find that the number of followers strongly influences repins. This is intuitive, since a user with more followers will have the pin routed to more feeds. Having a larger audience increases the likelihood of a repin, a common sense fact realized in our model. Furthermore, we find that activity level is negatively correlated with repins. The more pins a user posts, the less likely it is that her pins receive repins. The intiutive explanation for this observation is most likely represents the intuition that the more pins any user generates, the less likely any one of them is to be highly repinned.

### Color Effects

The remaining predictors in our model are related to colors. We are interested in quantifying the effect of visual features and their comparative effects on diffusion. We used a color variable that represents the dominant color of the image, as described previously.

We see from Table 3 that the highest effect on diffusion among image features belongs to color Red *β* = 0.49, *p* < 10^−15^. Purple (*β* = 0.31, *p* < 10^−15^) and Purple-Red (*β* = 0.16, *p* < 10^−15^) are also significant contributors to the number of repins. Note that Purple-Red is usually referred as Pink. White (*β* = 0.04, *p* < 10^−15^), Blue-Purple (*β* = 0.03, *p* < 10^−8^), Red-Yellow (*β* = 0.02, *p* < 10^−4^) and Green-Blue (*β* = −0.01, *p* < 10^−4^) have much smaller *β* coefficients, representing very small effect sizes.

Yellow *β* = −0.08, *p* < 10^−4^), Yellow-Green (*β* = −0.10, *p* < 10^−12^), Green (*β* = −0.13, *p* < 10^−8^), Black (*β* = −0.12, *p* < 10^−11^) and Blue (*β* = −0.17, *p* < 10^−12^), on the other hand, all negatively affect diffusion.

The overall results suggest that while the images with dominant color Red, Pink and Purple increase the chances of getting repins, while the images which are dominantly Black, Yellow, Yellow-Green, Green or Blue decrease the chances of diffusion.

We identify the images that are Black-and-White using a binary variable in our model. The model (Table 3) shows that when an image is Black-and-White, the chance of getting repins decreases (*β* = −0.12, *p* < 10^−8^). Saturation (*β* = 0.25, *p* < 10^−8^) has a large positive effect on repins, suggesting that highly saturated photos are more likely to be repinned on Pinterest. On the other hand, brightness (*β* = 0.02, *p* < 10^−12^) does not seem to impact diffusion.

## Discussion

In this work, we have taken a first step towards understanding how image content affects diffusion. We showed the effect of colors, brightness and saturation on the diffusion of pins on Pinterest. There has been extensive research on factors affecting virality in social media, but this is the first result we are aware of on the diffusion of images. In this section, we revisit and contextualize our results, offering implications for theory and practice, as well as points of departure for future work, both in design and theory.

### Images with color diffuse farther than those without

Our results show that there is an evident advantage in using color in images when it comes to spreading them. Black-and-White images are not shared as widely as colored images. This finding is in agreement with prior work where researchers found that magazine advertisements shown in color had a larger effect on readers than the Black-and-White ads [34, 35]. Because images that contain color are more likely to render the objects in the image more pleasing [64, 65], it follows that these images are more likely to be viewed more favorably (and as result shared more often) when they appear in color than Black and White [66]. Thus, under low processing motivation, users are more likely to be persuaded by images that make use of color, as it is the case with colored ads. Additionally a recent work by Meier and Robinson suggests that, when making evaluations, people automatically assume that bright objects are good, whereas dark objects are bad [67].

When the processing motivation is high, however, users are expected to engaged in more effortful processing, a hypothesis confirmed by studies on advertisements [68], allotting a sizable portion of their resource capacity to processing the image. Like less motivated users, the motivated viewers may initially attend to the photo; yet, they should go beyond this by processing the objects and content of the photo. The current study is focused more on the presentation of the photo as collection of pixels and what immediately appears to the viewer’s eyes.

### Red, Purple and pink drive diffusion

The major finding of this paper is that colors affect diffusion. This effect is large for several colors, including Red, Purple and pink. As we discussed, previous psychological studies confirm that Red confers [36, 38]. In a recent empirical work, Elliot et al. [39] focused on the influence of the color Red on performance in achievement situations. They posited that Red carries the meaning of failure in achievement situations and therefore evokes avoidance motivation in such situations. Although Red can have inimical implications for psychological functioning, it was shown that it leads men to view women as more attractive [as an aphrodisiac for men] because it carries the meaning of sex and romance in the context of heterosexual interaction [55].

Previous empirical work has supported the idea that Red has amorous meaning, as studies of color associations have indicated that people tend to connect Red to carnal passion, lust, and romantic love [4, 69–71]. In some of the earliest rituals known to anthropologists, Red ochre was used as face and body paint on females to symbolize the emergence of fertility [72, 73]. Red often appears as a symbol of passion, lust, and fertility in ancient mythology and folklore [74–78].

Mehta and Zhu [40] show that color can influence the task performance with Red working better with detailed-oriented task. Another study published in the journal Nature [54] shows that a similar effect can influence the outcome of physical contests in humans across a range of sports. They found that wearing Red is consistently associated with a higher probability of winning. These results indicate not only that sexual selection may have influenced the evolution of human response to colors, but also that the color of sportswear needs to be taken into account to ensure a level playing field in sports.

In animal experiments, some behavioral and morphological changes were reported by Salterelli and Coppola [79] when mice were exposed to Pink light (fluorescent light). Pink light, they reported, increased the weight of the adrenal compared to all other light conditions when the mice were exposed for 12 hours each day for a total of 30 days. In 1978, Schauss demonstrated the Kinesoid experiment, involving pink color, to a series of classes on innovative treatment techniques in corrections [80].

Our results confirm those findings, showing that images with a dominant color of Red, Purple and pink have higher chances of diffusion on Pinterest. In other words, images that contain Red and Purple are more likely to be shared by other users.

### Blue, Green, Yellow and Black suppress diffusion

We found that Blue, Green, Black, Yellow-Green and Yellow all negatively contribute to a pin’s diffusion. Interestingly, research suggests that both Blue and Green primarily have associations with calm and relaxing emotions. Yellow is a cheerful color, and Blue is associated with comfort and security [7]. One interpretation is that Pinterest users are more interested in sharing exciting (Red-colored images) and elegant (Purple colored images) images than cool, cheerful and relaxing ones (Blue, Green, Yellow). We need more work to discover *why* these colors matter and affect online communities this way. As we discuss later, drawing conclusions about underlying motivations and mechanisms in all observational and statistical work can be limited.

### High saturation drives diffusion

We found that a strong positive correlation exists between an image’s saturation and it’s degree of diffusion. Previous research has shown that highly saturated colors can be more exciting [41, 42, 46], as well as more widely liked [30, 43–45]. This is consistent with our findings.

### Implications

Our findings make a number of contributions to existing scholarship. Most importantly, we believe this work opens a new line of study: the present findings introduce new factors never before considered influential for diffusion. As we explain more fully next, we believe a deep thread of research can emanate from this work.

Our results connect to psychological studies of color and they highlight the importance of evocative content. Our work echoes the findings of earlier text-based studies: emotional activation is an important underlying diffusion driver within online social networks. In other words, we claim that online content that evokes specific emotions are often more viral. In color theory, Purple and Red are known as colors that elicit feelings of arousal (either in positive or negative direction), and our results reinforce this.

For practitioners rather than theorists, our findings shed light on how to construct viral content. Although we don’t claim that every image draped in Red or Purple will be shared significantly more, on average, warm and exciting colors seem to affect the recipient’s likelihood of sharing the image. Our results suggest that using warm, saturated colors can increase chances of diffusion compared to images with cool and relaxing themes.

Many mobile applications are providing users with photo-editing tools. One of the popular ways of editing photos is to apply filters to them. Filters can change saturation, brightness, and color distribution of the image. Our results can be used to design new filters for images. Filters that increase saturation or enhance the warmness of the image will likely increase engagement with the photo. In other words, a filter that saturates the image and adds Red tint will probably be better than a bluish one, in terms of virality.

### Limitations and Future Work

Pinterest is a product-oriented site with an emphasis on collecting and curating interesting content. Will the results be different for a professional photography site like Flickr, or a people-focused one like Instagram? We need more work to understand how site’s underlying uses and communities relate to the findings we have presented in this paper. For example, it seems reasonable to assume that on Imgur—a site designed for Reddit [81] and known to play host to many of that site’s memes—we would see a higher incidence of White, simply by accounting for the text overlaid on meme images. Conceivably, practices like this one could alter or shape the color findings we have presented.

Our work is purely quantitative and based on observational data. Our approach is useful for describing what factors affect diffusion, but without a corresponding qualitative approach, we can only speculate on why these factors matter. Further, the statistical methods we used examine only a small segment of behavior on Pinterest. Like many large-scale analyses over observational data, we cannot make any strong causal claims; future experimental work needs to corroborate these findings.

As we mentioned in the related work, Apter and his colleagues [28, 29] used a two dimensional theory to explain arousal and pleasure in color research. According to their theory, there are two dimensions of arousal, one positive, another one negative. Both excitement and tension can produce equivalently high states of arousal, but the former would be pleasant while the latter would be unpleasant [23]. When dealing with text sentiment analysis, we usually have two polarities of positive versus negative. An interesting area of future work is to investigate the different polarities of arousal caused by colors and their impact on online behavior.

An interesting research direction is the impact of context on color use. Is there a difference between Red food and Red clothes, in terms of virality? Another interesting follow-up direction is related to the interaction of colors in the image and how they affect diffusion. For example is there a difference between images having Red and Yellow, as compared to Red and Black?

We believe that our work is an initial step, and there is a rich landscape of research directions and open questions in this area. We see a set of research questions related to the other visual characteristics of multimedia and study their impact on online behavior. For example, what if we could use computer vision to relate macro-properties of an image (i.e., outside vs. inside, natural vs. cityscape) to how they diffuse? We know that people are naturally drawn to faces in offline life, is this also true online? It would be also worth considering demographic and cultural dimensions affect perceptions of color. Would our results hold for a site in which the user demographics are different from Pinterest? Finally, qualitative work can complement this work: What particular associations do people have with different colors, and what kinds of images seem to fall into these categories? For example, do primarily Orange images derive from natural photos or from manipulated ones?
